# Complementary Effects of Surgery and Pexidartinib in the Management of Patients with Complex Diffuse-Tenosynovial Giant Cell Tumor

**DOI:** 10.1155/2022/7768764

**Published:** 2022-12-03

**Authors:** Nicholas M. Bernthal, R. Lor Randall, Lauren N. Zeitlinger, Erik J. Geiger, John H. Healey

**Affiliations:** ^1^Department of Orthopaedic Surgery, David Geffen School of Medicine at UCLA, 1225 15th Street, Suite 2100, Santa Monica, CA, USA 90404; ^2^Department of Orthopaedic Surgery, University of California, Davis, 4860 Y Street, Suite 3800, Sacramento, CA, USA 95817; ^3^Department of Surgery, Orthopaedic Service, Memorial Sloan Kettering Cancer Center, 1275 York Avenue, New York, NY, USA 10065

## Abstract

Tenosynovial giant cell tumor (TGCT) is a rare neoplasm of the joint synovium that has a wide clinical spectrum including pain and stiffness in the affected joint, joint swelling, periarticular erosions, and cartilage loss, which can severely impact quality of life. The mainstay treatment for TGCT has been surgery involving partial or total synovectomy using arthroscopic or open techniques. However, surgical resection alone is associated with high recurrence rates, particularly in diffuse-TGCT (D-TGCT) cases. The 3 cases presented here summarize a combination approach (surgery+pexidartinib [tyrosine kinase inhibitor]) in patients with previously unresectable or inoperable D-TGCT. *Case 1-Hip.* A 29-year-old male was treated with pexidartinib prior to surgery, resulting in tumor reduction. A left total hip arthroplasty (THA) was then performed with a lack of recurrence in 12 months postoperative, and the patient currently on pexidartinib treatment. *Case 2-Foot.* A 35-year-old female, nearly a decade following a left foot mass resection, was treated with pexidartinib following disease recurrence. A decrease in soft tissue lesions at the midfoot and decreased marrow enhancement at the first metatarsal head were seen within 4–5 months of pexidartinib treatment; the patient is currently on pexidartinib (400 mg/day) with improved symptom control. *Case 3-Knee.* A 55-year-old male patient received pexidartinib pre- and postoperatively. A reduction in swelling and the size of the popliteal cyst was significant and maintained, with the synovial disease growing when pexidartinib was discontinued. Surgery and adjuvant therapy eliminated the disease as of the last follow-up visit (11 months postoperative). These cases provide a unique perspective based on tumor location, type/timing of treatment strategy, and patient outcomes. Optimal treatment strategies for this debilitating disease may entail utilizing a combination approach (surgery+systemic treatment) to reduce surgical morbidity and the risk of postoperative disease recurrence.

## 1. Introduction

Tenosynovial giant cell tumor (TGCT) is a rare, usually benign neoplasm derived from the synovium. TGCT affects joints, bursae, and tendon sheaths causing symptoms that include pain, inflammation, and joint stiffness [1–4]. This disease has a wide clinical spectrum capable of affecting patients of all ages but most commonly occurs in young adult females with a median age of 40 years [2, 5, 6]. TGCT is divided according to the site of origin (intra- or extra-articular) and growth pattern: localized TGCT (L-TGCT), formerly known as giant cell tumor of tendon sheath or diffuse TGCT (D-TGCT), previously referred to as pigmented villonodular synovitis (PVNS) [3, 7–9]. The worldwide incidence rate of TGCT (L-TGCT and D-TGCT combined) is estimated to be 43 per million, with the localized form being more prevalent than diffuse disease [10]. L-TGCT mainly affects small joints (i.e., fingers or wrists) [11], while D-TGCT frequently involves large joints such as the hip, ankle, elbow, or most commonly, the knee [12]. The mainstay of treatment for TGCT has been surgery involving partial or total synovectomy using arthroscopic or open techniques. While L-TGCT is often treated successfully with arthroscopic or open surgery incurring a low rate of recurrence, D-TGCT has been associated with high recurrence rates following both arthroscopy and open surgical approaches [1, 4]. Surgical treatment can result in increased morbidity and has a detrimental effect on patient quality of life [13, 14].

Recently, systemic treatment with tyrosine kinase inhibitors (TKIs) or monoclonal antibodies targeting the colony-stimulating factor-1 receptor (CSF1R), i.e., imatinib, nilotinib, emactuzumab, cabiralizumab, and pexidartinib, have been utilized with encouraging results in cases not amenable to surgery [15, 16]. The cases presented here demonstrate how CSF1R inhibitors (e.g., pexidartinib) in combination with surgery can be used effectively in patients with previously unresectable or inoperable D-TGCT. More specifically, we summarize multiple patient case studies in which pexidartinib was used prior to surgery, after surgery, or both to potentially downstage the extent of surgery, reduce surgical morbidity, or reduce the risk of disease recurrence postoperatively.

## 2. Case Presentations

### 2.1. Case 1-Hip

**Figure 1:**
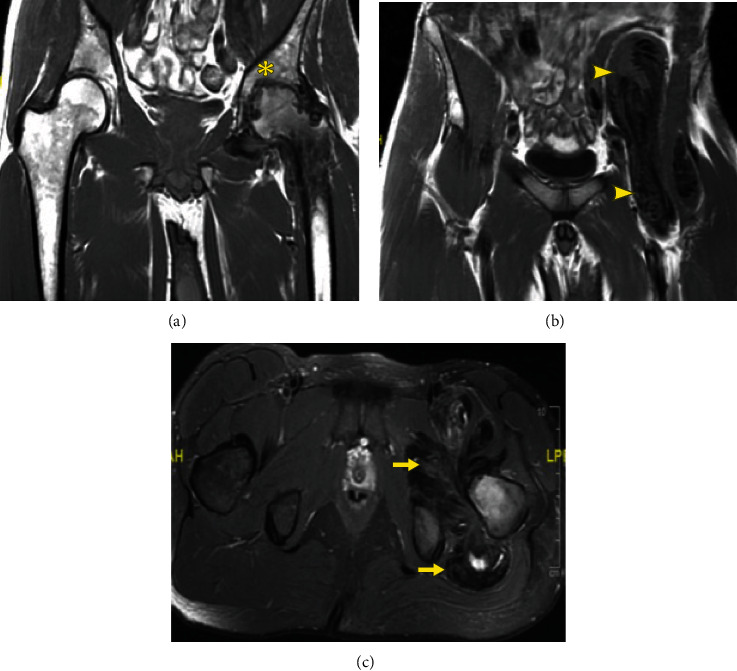
Representative MRI slices from of a 29-year-old male with D-TGCT in the left hip at diagnosis. Coronal (a, b) and axial (c) MR imaging demonstrating severe arthritic changes of the left hip joint with erosions present (^∗^), (b) massive TGCT tumor burden running along the anterior hip and into the pelvis adjacent to the iliacus muscle (arrowhead), and (c) tumor burden both posterior to the hip in the ischiofemoral fossa and anterior to the psoas insertion (arrow).

A 29-year-old male with no past medical history developed new onset left-lateral hip pain while running in August 2019. After an unsuccessful trial of physical therapy, an MRI was obtained in December 2019 that showed a lobulated mass anteriorly and posteriorly around the left hip extending into the left pelvis anteromedial to the iliacus musculature and posteriorly into the ischiofemoral space (Figures 1(a)–1(c)). A representative measurement performed at the level of the external iliac vessels measured 7 cm × 3 cm × 7 cm. There was advanced left hip arthritis with severe joint space narrowing, subchondral sclerosis, cartilage loss, and osteophytic spurring (Figures 1(a)–1(c)).

**Figure 2:**
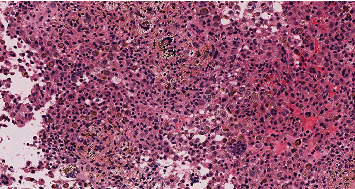
Histology images in the hip prior to pexidartinib treatment. The pretreatment biopsy demonstrating a cellular proliferation of tumor cells composed of abundant hemosiderin laden macrophages, multinucleated giant cells, scattered foam histiocytes, and lymphocytes (H&E 200X).

After image-guided biopsy, histology of the neoplasm was consistent with D-TGCT. Sections of the tumor from the pretreatment biopsy demonstrated a cellular lesion composed of mixed inflammatory cells including macrophages with hemosiderin pigment and abundant multinucleated giant cells, as well as collections of foamy histiocytes and lymphocytes (Figure 2). In January 2020, 1 month after initial imaging, the patient started neoadjuvant pexidartinib 200 mg twice daily with a goal of tumor volume reduction to facilitate surgical resection and total hip arthroplasty to address the severe arthritis. A repeat MRI in February 2020 demonstrated a slight interval decrease in the size of the mass: measurement at the level of the external iliac vessels was 5.6 cm × 3.0 cm, with minimal change posteriorly. His hip pain was stable, for which he used daily diclofenac. Four months later (June 2020), the pexidartinib dose was increased to 400 mg twice daily (morning and evening), which he tolerated well until an episode of shingles in September 2020, at which point the drug was held. Pexidartinib was restarted at the same dosing 1 week later after resolution of the pruritic rash. At this point, the patient still experienced intermittent left hip pain while walking and was taking anti-inflammatory pain medication.

**Figure 3:**
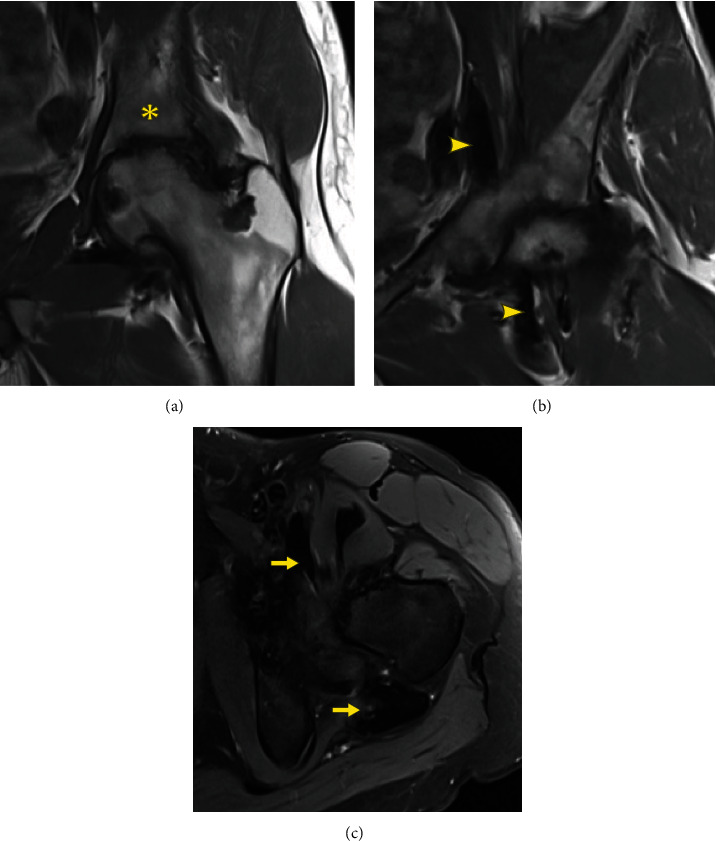
Representative MRI slices of a 29-year-old male with D-TGCT in the hip 12 months after the initiation of pexidartinib. Post-pexidartinib treatment coronal (a, b) and axial (c) MR imaging demonstrates persistent end stage hip arthritis (^∗^) but significant reduction in TGCT tumor burden tracking along the anterior hip into the pelvis (arrowhead) as well as significant reduction of both the ischiofemoral and anterior psoas tumor burden (arrow).

At a follow-up visit in December 2020, MRI revealed a reduction in tumor size, with representative tumor measurements performed at the level of the external iliac vessels of 5.0 cm × 2.1 cm and at the ischiofemoral space of 4.3 cm × 2.0 cm (Figures 3(a)–3(c)), specifically involving the iliopsoas bursa and ischiofemoral space. The patient continued taking diclofenac once or twice daily for pain management.

**Figure 4:**
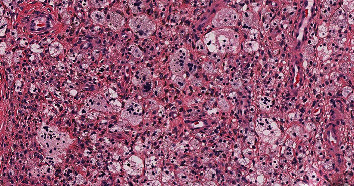
Histology images in the hip post-pexidartinib treatment. The posttreatment specimen was less cellular compared to the pretreatment tissue and demonstrated a relative increased number of foamy histiocytes and significantly decreased number of multinucleated giant cells (H&E 200X).

In April 2021, a left total hip arthroplasty (THA) was performed successfully though a posterior approach, resecting all accessible tumors. A 6 cm × 6 cm × 5 cm infiltrative brown specimen was removed during the posterior approach to the hip capsule, including an additional tumor that was removed from around the posterior acetabulum; severe arthritic changes to the hip joint were observed. Histologic review of the resected specimen (post-pexidartinib treatment) demonstrated a mixed population of cells. However, compared to the pretreatment sample, the tissue was less cellular, the foamy histiocytic component was increased, and the number of multinucleated giant cells were significantly decreased (Figure 4).

**Figure 5:**
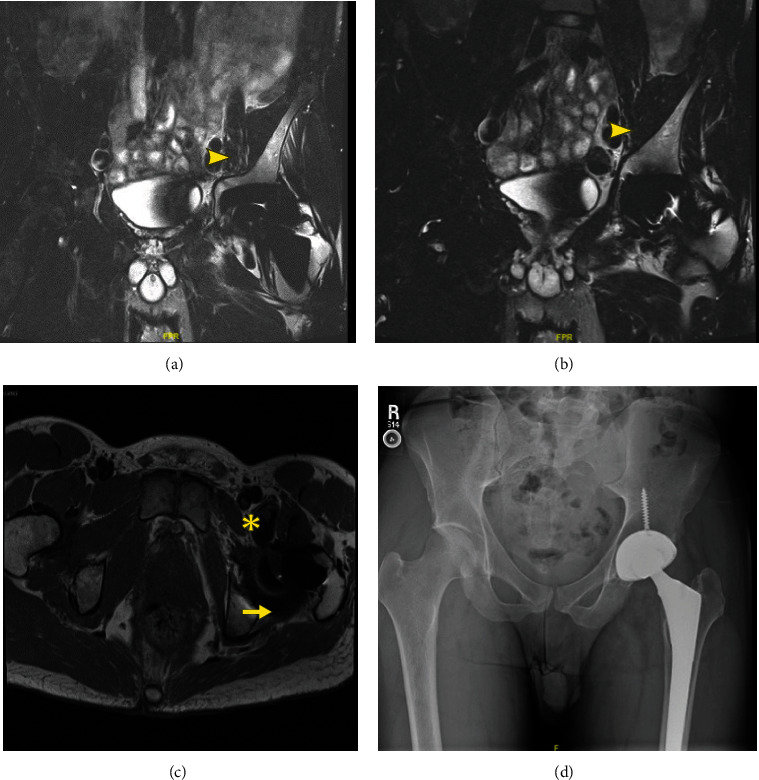
Representative MRI slices and postoperative radiographs of a 29-year-old male with D-TGCT in the left hip. Coronal MRI with metal subtraction (a, b) demonstrates near resolution of the intrapelvic tumor burden (arrowhead). Axial imaging (c) demonstrates absence of posterior tumor burden after surgical resection (arrow) but persistence of the small anterior tumor along the psoas muscle (^∗^). A well-fixed cementless total hip arthroplasty is demonstrated (d).

The anterior pelvic disease was not pursued at the same time given the elevated risks of hip instability and infection associated with dual surgical approaches. Neoadjuvant pexidartinib treatment (400 mg twice daily) was resumed the following month (May 2021) to treat the residual anterior intrapelvic disease. At the last orthopedic follow-up in April 2022, the patient had no hip pain, was ambulating unassisted, and exercising comfortably. Follow-up X-rays demonstrated a well-sized and fixed cementless total hip arthroplasty (Figures 5(a)–5(d)). Surveillance MRI at the same time demonstrated resection of the tumor from the ischiofemoral space with slight shrinkage of the anterior tumor along the iliac vessels (now 4.3 cm × 1.1 cm) (Figures 5(a)–5(d)). Currently, the patient remains on pexidartinib, with the only side effect being hair color change.

### 2.2. Case 2-Foot

The patient was a 35-year-old female with no past medical history who initially presented in 2008 at age 22 with foot swelling and pain. A radiograph in 2009 showed multiple low-signal intensity masses with diffuse heterogeneous enhancement about the joints of the midfoot extending approximately 5.8 cm over the dorsal aspect of the foot from the base of the metatarsals over the head of the talus. Along the dorsal aspect of the navicular bone, the mass measured approximately 9 mm medially. The thickest portion of the mass dorsally measured approximately 1.4 cm overlying the cuneiforms.

In January 2010, the patient accepted surgery, and a resection of the left foot mass was performed. Nearly 9 years later (December 2018), the patient was symptomatic again with foot swelling and pain, and radiographs showed osseus destructive changes of the first, second, and third cuneiforms and cuboid, representing recurrent disease. In addition, a large bulky soft tissue mass associated with the fourth and fifth metatarsal was observed as 3.78 cm proximal-distal, 1.13 cm medial-lateral, and 2.5 cm dorsal-plantar. The mass was at low-signal intensity on T1 and T2, compatible with D-TGCT. At that point, the patient was considered a poor candidate for surgery, declined systemic treatment (imatinib and pexidartinib), and received three cortisone injections directly into the lesion between March 2019 and September 2019.

**Figure 6:**
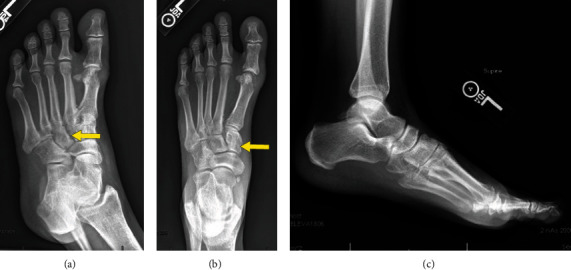
Representative images of a 35-year-old female with D-TGCT in the foot. Large erosion at the medial head of the first metatarsal, and throughout the midfoot, consistent with reported history of D-TGCT from an (a) oblique (arrow), (b) anterior-posterior (arrow), and (c) lateral view with multiple sites of midfoot erosions.

In March of 2019, a core needle biopsy showed D-TGCT. Four months later at a follow-up visit in July 2019, an ill-defined soft tissue mass with associated erosions in the midfoot measuring 3.0 cm × 2.0 cm × 2.8 cm was observed in the radiographs (Figures 6(a)–6(c)).

**Figure 7:**
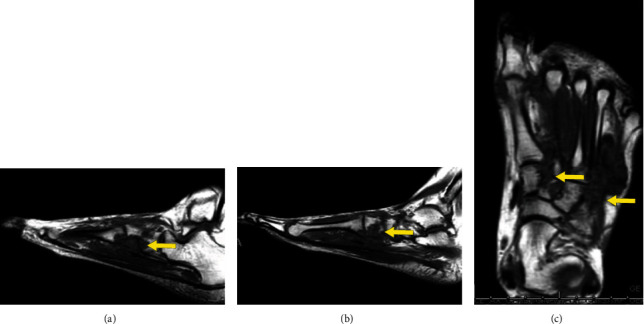
Sagittal and axial images of a 35-year-old female with D-TGCT in the foot. Soft tissue masses at the medial margin of the first metatarsal head and centered about the first through fourth tarsometatarsal joints and extending to the navicular bone appear unchanged, with accompanying bony erosions. These demonstrate low signal on T1 images, compatible with provided history of diffuse tenosynovial giant cell tumor. Low-signal mass dorsal to the fourth tarsometatarsal joint: (a, b) T1 weighted sagittal images of the foot (arrow) and (c) T1 weighted axial view of the foot (arrow).

In September 2019, the patient had an Eastern Cooperative Oncology Group performance status score of 0 and was able to work with pain while taking naproxen. One month later, radiographs showed an erosion with associated edema and enhancement involving the medial first metatarsal head, and gout is also within the differential; similar-appearing erosions with associated soft tissue mass involving the midfoot were also observed. The mass measured 3.0 cm in the anterior-posterior dimension and 2.0 cm × 2.8 cm in transverse dimension (Figures 7(a)–7(c)).

In October 2019, pexidartinib was started at 400 mg daily. After 11 days of treatment, pexidartinib was held for nearly 3 weeks due to a grade 3 rash and then restarted at 200 mg daily. A week later, pexidartinib was increased to 400 mg daily (for 9 days) and 600 mg daily thereafter.

**Figure 8:**
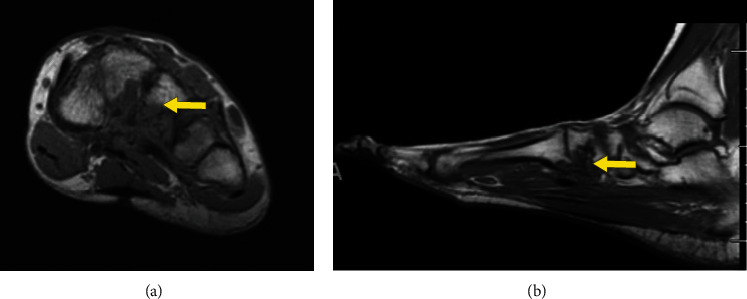
Coronal and sagittal images of a 35-year-old female with D-TGCT in the foot. (a) Coronal and (b) sagittal T1 weighted images demonstrating overall stable appearance of the hypointense T1 weighted images of the foot (arrow), consistent with known D-TGCT.

In March 2020 at a follow-up visit, an MRI (Figures 8(a) and 8(b)) showed decreased enhancement and volume of TCGT soft tissue lesions at the midfoot and decreased marrow enhancement at the first metatarsal head. It is difficult to measure given the osseous erosive changes and extensive nature of the mass, but overall, it appeared with decreased enhancement and size. One week later, pexidartinib was decreased to 400 mg daily due to cognitive issues.

**Figure 9:**
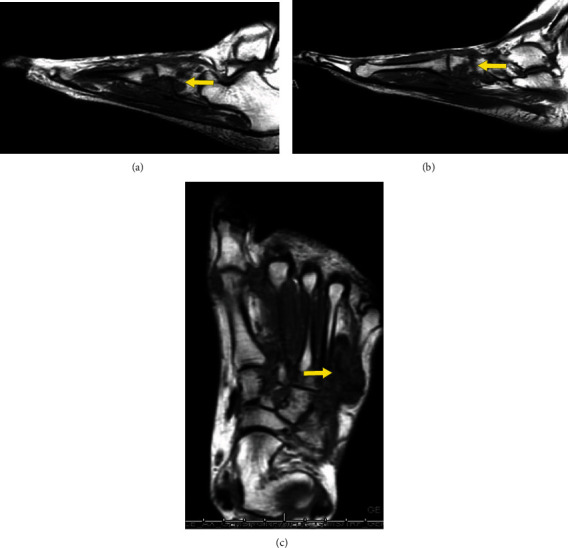
Sagittal and axial images of a 35-year-old female with D-TGCT in the foot. Sagittal T1 weighted images (a, b) (arrow) and axial T1 weighed image (c) (arrow) demostrating stable involvement of the forefoot and midfoot with known history of D-TGCT on serial imaging.

In January 2021, the MRI (Figures 9(a)–9(c)) showed no change in the appearance of multifocal masses about the left midfoot and adjacent to the first metatarsal head relative to the preceding MRI from March 2020, compatible with TGCT.

Currently, the patient continues to have waxing and waning side effects and has been able to decrease the cognitive impairment by intermittently holding the dose. The dose of pexidartinib is back up to 400 mg daily for improved symptom control, and the dose is being titrated based on symptoms and side effect profile.

### 2.3. Case 3-Knee

**Figure 10:**
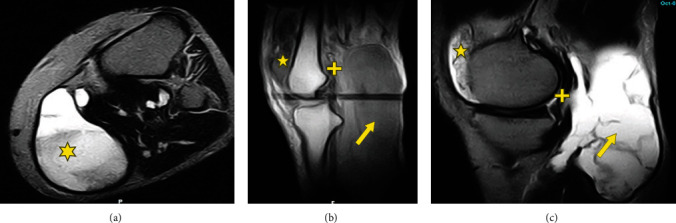
Axial and sagittal images of a 55-year-old male with D-TGCT in the knee. (a) MRI with axial T2 fat suppressed images of the proximal lower leg. The large posteromedial hyperintense mass (star) shows a fluid-fluid level from the synovial fluid in the anterior aspect and proteinaceous and bloody fluid posteriorly. (b) MRI sagittal T1 view of the knee. Anteriorly, (star) the suprapatellar pouch is filled with an intermediate intensity fluid and contains diffuse hypointense material characteristic for the solid component of tenosynovial giant cell tumor. Posterior to the joint, there are multiple aggregations of hypointense masses along the posterior capsule and origins of the gastrocnemius muscles and the posterior cruciate ligament. Posteriorly, (arrow) the enormous popliteal cyst is evident with intermediate intensity. (c) MRI with sagittal T2 fat suppressed images of the knee. This shows the same fluid filled suprapatellar pouch with diffuse areas of lower intensity scattered throughout (star). Diffuse dark bands of thickened posterior capsule and tumor extend outside the joint and behind the posterior cruciate ligament (plus). The large hyperintense fluid filled popliteal cyst with scattered hypointense bands and nodules are present posteriorly (arrow).

A 55-year-old male patient initially presented 2 years prior (at age 53 in 2017) with painful knee swelling and radiographs showing loose bodies. The patient was treated by aspirations of the cyst followed by subsequent cortisone injections, resulting in short-term improvement. The cyst returned and was more symptomatic with increased stiffness and pain in 2019. In October 2019, a left knee MRI showed a large fluid collection, a Baker's cyst (13 cm), and a moderate effusion that was partially ruptured inferiorly. Three weeks later, a left knee arthroscopy with total synovectomy confirmed D-TGCT (Figures 10(a)–10(c)). A popliteal cyst measuring 13 cm × 6 cm × 5 cm with unquantified diffuse thickening of the anterior and posterior synovium was observed in the radiographs.

At a follow-up visit in June 2020, radiographs revealed persistent D-TGCT that increased, surrounding the cruciate ligaments. A 3.4 cm × 2.0 cm mass in the medial gastrocnemius, doubling of the thickened synovium around the cruciate ligaments, tibial articular erosions, and mild degenerative changes were observed, in addition to a slight reduction of the popliteal cyst, which measured at 11.5 cm × 5.6 cm × 0.4 cm.

**Figure 11:**
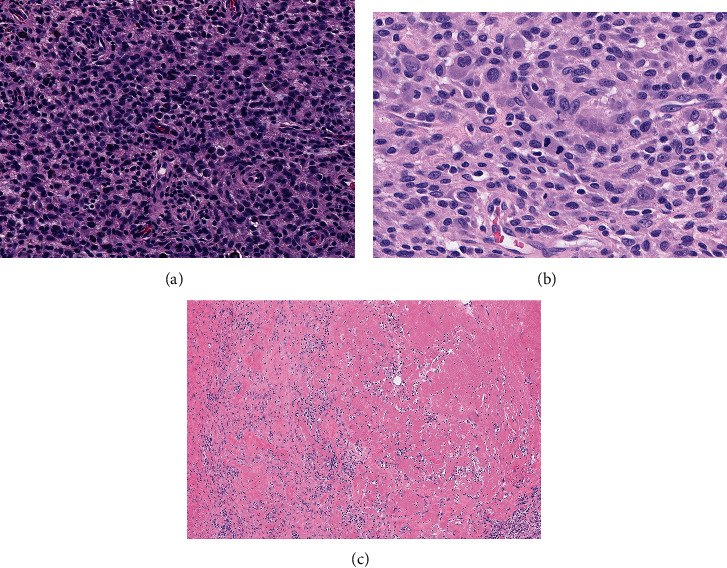
Histology images in the knee post-pexidartinib treatment. Hypercellular region of the popliteal tumor after pexidartinib treatment is indistinguishable from that seen in the original biopsy (a). This photomicrograph displays areas of highly mitotic proliferative tumor (19 mitoses per 10 high power fields) that were widely seen in the viable tumor (b). Approximately, 30% of the mass was composed of these areas of fibrotic hypocellular tissue with low proliferative rates (c).

**Figure 12:**
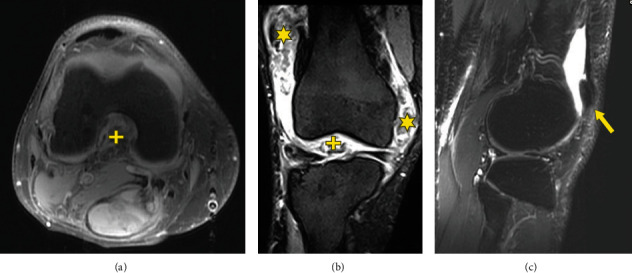
Axial, coronal, and sagittal images of a 55-year-old male with D-TGCT in the knee. MRI shows tumor measuring 2.8 cm × 1.4 cm was in the medial and lateral gutters (star) along with tumor in the intercondylar notch posteriorly (plus). There were also synovial thickened nodules extending into the popliteal cyst, which shrank to 5.8 cm and contained hypointense debris and hemorrhage (arrow). (a) An axial fat suppressed image of the distal femur, (b) a coronal T2 fat suppressed image of the knee, and (c) a sagittal T2 fat suppressed image of the lateral aspect of the knee joint that best demonstrated the residual tenosynovial giant cell tumor (arrow).

One month later (July 2020), the patient started on pexidartinib at 200 mg twice daily, which resulted in reduced pain and swelling. Treatment was halted after 5 weeks due to grade 4 neutropenia. Sections from the histological examination of the resection specimen show that the hypercellular region of the popliteal tumor (Figure 11(a)) is similar to what was observed prior to pexidartinib treatment. In addition, areas of highly mitotic proliferative tumor were present (Figure 11(b)), and nearly one-third of the tumor was made up of fibrotic, hypocellular tissue with low proliferative rates (Figure 11(c)). In October 2020, MRIs showed lesions measured at 2.8 cm × 1.4 cm in the medial gutter and posterior intercondylar notch (Figures 12(a)–12(c)). Synovial thickening extended into the popliteal cyst, which shrank to 5.8 cm, containing debris and hemorrhage.

The tumor grew, and symptomatic swelling, stiffness, and pain increased while awaiting hematologic normalization. White blood cell counts were restored, and surgery was performed in December 2020. Specifically, the left posterior knee synovectomy showed D-TGCT (9.7 cm × 7.5 cm × 4.3 cm in aggregate). The tumor showed areas of increased cellularity and increased mitotic activity (up to 19 mitoses/10 hpfs). Areas of fibrosis and necrosis were consistent with therapy-related changes (estimated as 30% of the mass). The left anterior knee synovectomy also showed D-TGCT (10.0 cm × 9.0 cm × 3.0 cm in aggregate) with focal areas of fibrosis consistent with therapy-related changes (~10%). There was no recurrence following surgery, and follow-up radiographs from January 2021 did not show a tumor.

The next month (February 2021), the patient restarted adjuvant therapy with pexidartinib at 200 mg twice daily for a planned 2 months. At a follow-up visit in April 2021, radiographs demonstrated extensive postoperative changes and edema but no recurrence of TGCT. No tumor was diagnosed; however, a small area along the lateral tibial plateau was reported as scar, blooming effect, representing postoperative metal artefactual particles or focal residual disease.

**Figure 13:**
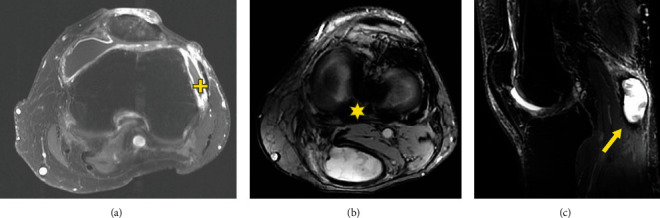
Effusion in the knee of a 55-year-old male with D-TGCT. No tumor recurrence is observed following rupture of anterior cruciate ligament. (a) This axial view with contrast of the distal femur shows a suprapatellar pouch effusion with hyperintense capsular enhancement (plus). No tumor is visible. (b) The axial view at the level of the menisci has areas of hypointensity interpreted by the radiologist as scar rather than tenosynovial giant cell tumor along the posterior cruciate ligament insertion (star). (c) This sagittal view shows a dramatic reduction of the popliteal cyst with small residual nodules of debris in the cyst (arrow).

In October 2021, the patient suffered a tear of the anterior cruciate ligament, and radiographs showed effusion plus surgical changes around the posterior tibial border. A new rupture of the anterior cruciate ligament and thinning of the posterior cruciate ligament were observed; there was no evidence for recurrent TGCT (Figures 13(a)–13(c)).

## 3. Discussion

In cases in which surgery is contraindicated or presents a high morbidity risk, or those in which the tumor is unresectable, systemic treatment can be employed with the goal of reducing the size of the tumor and improving these patients' symptoms and quality of life. In an additional treatment pathway illustrated by these case examples, systemic treatment can also be utilized as a complementary treatment method to downstage the tumor turning patients who had unresectable disease into reasonable surgical candidates; suggesting a multidisciplinary approach can be beneficial in treatment of D-TGCT.

Pexidartinib is a selective CSF1R inhibitor that targets the CSF1/CSF1R pathway involved in the pathogenesis of TGCT [17]. Administration of pexidartinib has resulted in significant tumor reduction in patients with TGCT [17, 18]. Subsequently, it was approved by the U.S. Food and Drug Administration and later designated by the National Comprehensive Cancer Network as a category 1 recommendation for adult patients who have symptomatic TGCT and severe morbidity or functional limitations and for whom surgery is not an option [19–21]. Pexidartinib-treated patients have also demonstrated significantly better patient-reported outcomes (i.e., greater mobility and improvement of worst stiffness) [18]. After careful evaluation, if systemic therapy (i.e., pexidartinib) is the treatment of choice, we suggest that a reevaluation for surgical candidacy should be performed in 3–6 months following the start of treatment. If there has been no interval improvement in or a progression of symptoms, then attempted surgical resection should be considered. However, a patient can continue systemic treatment if symptomatic improvement (pain, swelling, stiffness, and range of motion) has been achieved, if the tumor remains unresectable, and if the drug is tolerable. The combination of neoadjuvant pexidartinib therapy and surgical resection can be implemented to reduce the likelihood of recurrence in cases either having a large volume of disease or having anatomic constraints that preclude complete tumor resection [22]. In the ENLIVEN study, 32/61 (52.4%) of pexidartinib-treated patients had received ≥1 prior surgery [18]. Treatment with pexidartinib has been associated with severe adverse reactions (e.g., hepatotoxicity) [18, 23]; optimal duration of use remains unclear. The approval of pexidartinib was conditional on its prescription via a mandatory REMS due to serious and potentially fatal liver injury [24]. As such, and to mitigate potential risks, the three patients summarized here started at a daily dose of 400 mg pexidartinib which is half of the approved dose of 800 mg daily [19]. It is critical to weigh the risks and benefits of drug treatment and closely monitor patients on the drug in cases in which dose reduction or discontinuation becomes necessary [22]. As demonstrated in the 3 cases reported here, pexidartinib treatment was effective in decreasing tumor size and improving symptoms, dose reductions and temporary discontinuation of the drug occurred due to adverse reactions, and highlighting the flexibility that can be used in dosing the drug based on the clinical response and how well the patient tolerates the drug.

In our cases, pexidartinib was administered in combination with surgery at different stages (neoadjuvant, adjuvant, or both). For the case in which D-TGCT was presented in the hip, the patient was treated with pexidartinib prior to surgery, resulting in a reduction of tumor volume size. Thereafter, a successful THA was performed to remove all the accessible tumors and address the severe arthritis. As of the last follow-up visit, there remains a lack of recurrence (currently 11 months postoperative), and the patient is currently on pexidartinib treatment.

Regarding the patient who presented with disease in the foot, nearly a decade following surgery (resection of the left foot mass), the disease recurred and was treated with pexidartinib, as surgery would result in midfoot fusion and high likelihood of postsurgical morbidity. A high rate of complication and recurrence has been observed in the foot and ankle [3]. For this case, given the osseous erosive changes and the extensive nature of the mass, it was difficult to measure changes in the tumor; however, a decrease in soft tissue lesions at the midfoot and decreased marrow enhancement at the first metatarsal head were observed within 4–5 months of pexidartinib treatment initiation. The patient is currently on 400 mg/day pexidartinib with improved symptom control, and the dose is titrated based on side effects.

For the case showing D-TGCT in the knee, the patient received pexidartinib prior to surgery and postoperatively. It was challenging to measure the extent of the disease due to multiple contributors, including a complex popliteal cyst with debris and the presence of degenerative arthritis changes. Episodic treatment is possible, and responses in the neoplastic and inflammatory components of the disease can be observed and may be discordant. A reduction in swelling and the size of the popliteal cyst was remarkable and sustained due to reduction of the inflammatory aspect of the disease, with the synovial disease growing when drug therapy was discontinued. Ultimately, the surgery and adjuvant therapy eradicated the disease as of the last follow-up (October 2022). The patient was seen in October 2022, responding to physical therapy with greater strength and range of motion, less pain, and no documented recurrence on a new MRI.

After baseline intervention, these cases were followed with clinical and radiological MRI follow-up occurring approximately 3 months later. This allowed for evaluation of the success of therapy and offered a reference point for additional follow-ups. Thereafter and in accordance with current recommendations [3], MRI follow-up visits occurred approximately every 6 months, which was sufficient for detection of any potential recurrences. Exceptions to these scheduled visits are dependent upon specific clinical presentations and/or patient complaints as observed in these cases, with specific symptoms and adverse reactions occurring with systemic treatment.

The optimal treatment strategy in patients with D-TGCT is currently evolving. As surgery can result in a high recurrence rate and is associated with surgical morbidity [10], utilization of systemic therapy could be beneficial in certain cases. Each of these cases provides a unique perspective based on tumor location, type, timing of treatment strategy (systemic treatment before and/or after surgery), and patient outcomes with this debilitating disease. As these cases demonstrate, the best management of patients with D-TGCT involves a multidisciplinary therapeutic approach, which is most efficiently provided at sarcoma referral centers that will see a larger volume of patients with this rare disease.
